# Tunlametinib Suppresses Melanogenesis Induces Cell Cycle Arrest and Apoptosis in Giant Congenital Melanocytic Nevus Cells

**DOI:** 10.3390/cancers18193166

**Published:** 2026-10-01

**Authors:** Jin-Ye Guan, Zhuo Chen, Wen-Shang Liu, Jia-Long Xu, Zhi-Yu Zhou, Lina Song, Dan Deng

**Affiliations:** Department of Dermatology, Shanghai Children’s Medical Center, School of Medicine, Shanghai Jiao Tong University, Shanghai 200127, China; 123731910806@sjtu.edu.cn (J.-Y.G.); chenzhuo@scmc.com.cn (Z.C.); sixwinsong@163.com (W.-S.L.); xjl19204947320@163.com (J.-L.X.); zhiyu_zhou@126.com (Z.-Y.Z.)

**Keywords:** giant congenital melanocytic nevus (GCMN), tunlametinib, MEK inhibitor

## Abstract

**Background/Objectives:** Giant congenital melanocytic nevus (GCMN) is a rare developmental disorder arising from abnormal proliferation and differentiation of neural crest–derived melanocytes. It poses substantial clinical challenges due to cosmetic disfigurement, malignant potential, and the limited efficacy of current treatments. This study aimed to investigate the therapeutic effects and underlying molecular mechanisms of tunlametinib (HL-085), a novel selective MEK1/2 inhibitor, in GCMN. **Methods:** Primary GCMN cells and human GCMN explants were isolated, cultured, and validated to establish in vitro cellular and explant models. Following tunlametinib treatment, global transcriptional changes were analyzed using bulk RNA sequencing. Melanogenesis was assessed via Fontana–Masson staining, Western blotting, and immunofluorescence. Cell viability, apoptosis, and cell cycle distribution were evaluated using the CCK-8 assay, flow cytometry, TUNEL staining, and Western blotting for apoptosis- and cell cycle–related proteins. **Results:** Tunlametinib treatment significantly suppressed melanogenesis in GCMN cells and explant tissues at the phenotypic, histological, and protein levels, and reduced cell viability in a concentration-dependent manner. Transcriptomic analysis revealed marked alterations in gene expression profiles following treatment, with prominent changes in apoptosis- and cell cycle–related genes. Subsequent cellular assays and tissue staining confirmed that tunlametinib significantly increased early apoptosis and induced G1/S cell cycle arrest. **Conclusions:** Collectively, our findings demonstrate that tunlametinib inhibits proliferation and melanin production in GCMN cells by modulating the RAS-ERK pathway, providing a theoretical basis for its use as a targeted therapeutic strategy for GCMN.

## Simple Summary

Giant congenital melanocytic nevus is a rare skin condition present at birth and caused by the abnormal growth of pigment-producing cells. Patients may experience large dark skin lesions, cosmetic and psychological burden, and a risk of developing melanoma. However, effective non-surgical treatments remain limited. In this study, we explored whether tunlametinib, a targeted drug that blocks a key growth-related signaling pathway, could reduce pigment production and inhibit the growth of cells derived from patients with giant congenital melanocytic nevus. Using patient-derived cells and cultured tissue samples, we found that tunlametinib reduced melanin production, suppressed cell growth, promoted cell death, and blocked cell cycle progression. These findings suggest that tunlametinib may represent a potential targeted treatment strategy and provide useful experimental evidence for future research on giant congenital melanocytic nevus and related melanoma risk.

## 1. Introduction

Giant congenital melanocytic nevus (GCMN) is a rare developmental disorder arising from abnormal proliferation and differentiation of neural crest–derived melanocytes. Clinically, GCMN is present at birth and is characterized by extensive lesion size with frequent involvement of deep dermal structures. In a subset of cases, the condition may extend to the central nervous system [1]. Beyond its substantial impact on appearance and quality of life, GCMN is associated with an increased risk of malignant transformation. In a 20-year single-center retrospective study of 152 patients with GCMN, malignant tumors developed in around 5% of patients, including four cases of melanoma [2]. A subsequent systematic review including 7915 patients with congenital melanocytic nevi reported 91 melanomas and found that, by 15 years of age, the melanoma incidence density in patients with large-to-giant CMN was approximately 21.9-fold higher than that in patients with small-to-medium CMN [3]. Melanoma may develop within associated melanocytic proliferations and can also involve extracutaneous sites, including the central nervous system, particularly in patients with extensive GCMN or neurocutaneous melanocytosis [4]. Neurocutaneous melanocytosis is an uncommon but potentially severe complication associated with extensive congenital melanocytic nevi [5]. Current clinical management relies predominantly on surgical excision, skin grafting, and laser-based approaches. These interventions may reduce nevus burden and could potentially influence the subsequent risk of melanoma by decreasing the number of nevus cells at risk for malignant transformation. However, the large lesion area, deep tissue involvement, and high incidence of postoperative scarring or recurrence often limit the efficacy of these interventions [6,7]. More importantly, these interventions are largely symptomatic and fail to address the molecular drivers of disease initiation and progression. Consequently, the development of safe, mechanism-based targeted therapies remains a major unmet clinical need in the treatment of GCMN.

At the molecular level, genetic studies have established that activating NRAS mutations represent one of the most common driver events in large and giant congenital melanocytic nevi, with mutations at codon 61 being particularly prevalent [1,8]. However, GCMN is genetically heterogeneous, and not all lesions are driven by NRAS mutations, suggesting that additional genetic alterations may also contribute to disease pathogenesis. Nevertheless, NRAS Q61 mutations remain among the best-characterized molecular alterations in GCMN. Previous studies have demonstrated that this mutation renders NRAS constitutively active, leading to persistent activation of the downstream RAF–MEK–ERK (MAPK) signaling pathway. Sustained MAPK signaling aberrantly promotes melanocyte proliferation, enhances cell survival, and disrupts normal terminal differentiation, thereby driving clonal expansion and accumulation of mutant melanocytes, ultimately contributing to the development and progressive enlargement of GCMN. This mechanistic insight provides a strong rationale for targeting the MAPK pathway in GCMN. Notably, pharmacological inhibition of RAS–MAPK signaling has been shown to effectively suppress GCMN-associated cellular phenotypes in both in vitro and in vivo models [9,10]. In addition, emerging clinical observations suggest that MEK inhibitors may alleviate severe GCMN-related symptoms, such as pruritus, pain, and local swelling, further supporting the therapeutic potential of MAPK-targeted interventions [11].

Clinically, MEK inhibitors first demonstrated therapeutic efficacy in *BRAF*^V600E^-mutant melanoma, particularly in combination with BRAF inhibitors, where they delay resistance and improve survival [12]. Their application has since expanded to MAPK-driven developmental disorders and benign proliferative diseases, such as neurofibromatosis type 1, low-grade glioma, and congenital pigmentary disorders [1,13,14]. In GCMN, current evidence remains preliminary but promising. Preclinical studies have shown that trametinib and binimetinib suppress MAPK activation, inhibit nevus cell proliferation, and may be suitable for local intradermal delivery [9,10], while early clinical reports suggest rapid improvement of both symptoms and lesions in *BRAF* fusion-driven GCMN treated with trametinib [11,15,16]. These findings support MEK inhibitors as a potential MAPK- and genotype-guided precision therapy for GCMN, although larger clinical studies are needed to determine their efficacy, safety, and optimal use. Although MEK inhibitors have shown biological activity in GCMN, current evidence remains largely limited to case reports and preclinical models, and complete lesion regression is rarely achieved, which may reflect multiple factors, including residual nevus cell survival, limited tissue penetration, pathway redundancy or compensatory signaling, and genetic heterogeneity [17]. Moreover, the need for long-term systemic therapy in a pediatric, non-malignant setting poses substantial concerns regarding tolerability and safety. These limitations highlight the necessity of evaluating more selective and better-tolerated MEK inhibitors with favorable pharmacokinetic profiles for *NRAS*-driven GCMN.

Tunlametinib is a novel, ATP-noncompetitive MEK inhibitor and the first domestically developed MEK-targeted agent specifically designed for NRAS-mutant melanoma [18]. Preclinical studies have shown that tunlametinib potently inhibits MEK activity with minimal off-target kinase inhibition, indicating high target selectivity. Favorable pharmacokinetic properties and tolerability profiles have been reported in early-phase clinical studies, and therapeutic activity has been demonstrated across multiple RAS- or RAF-driven malignancies, including melanoma, non-small cell lung cancer, and colorectal cancer [19,20]. A recent case report described a pediatric melanoma arising from an NRAS-mutant GCMN that showed rapid tumor progression despite surgery, chemotherapy, and immunotherapy [21]. Subsequent treatment with HL-085 provided transient disease control and survival benefit, but its efficacy was limited by high proliferative activity, multilineage *NRAS* heterogeneity, delayed treatment initiation and dose adjustments. This case highlights the potential value of HL-085 in *NRAS*-mutant malignant transformation of GCMN, while the optimal timing, dosing strategy, resistance mechanisms, and combination regimens in pediatric patients require further investigation. Given the shared activation of the RAS–MAPK pathway in melanoma and a substantial subset of GCMN, HL-085 may represent a potential treatment option for GCMN. In NRAS-mutant melanoma, phase I and II studies have demonstrated that HL-085 exhibits acceptable tolerability, favorable pharmacokinetic properties, and clinically meaningful antitumor activity [19,20,22], providing pharmacological support for targeting NRAS-driven MAPK signaling with this agent.

However, the effects of tunlametinib on the biological behaviors of GCMN cells, including pigmentation, proliferation, apoptosis, and cell-cycle regulation, remain poorly understood. In this study, we evaluated the effects of tunlametinib on primary GCMN cells derived from both NRAS-mutant and non-NRAS-mutant patients and characterized its impact on cellular proliferation, apoptosis, cell-cycle distribution, and pigmentation-related changes. By integrating functional assays, protein-level analyses, and transcriptomic profiling, this study provides a comprehensive preclinical evaluation of the biological effects of tunlametinib in GCMN cells and offers preliminary molecular insights supporting further investigation of MEK inhibition as a potential therapeutic strategy for this disease.

## 2. Materials and Methods

### 2.1. Cell Culture

GCMN cells were isolated from human GCMN tissue obtained from three independent patients. Lesion tissue was obtained at the time of surgical intervention, from patients admitted to the dermatology department of Shanghai Children’s Medical Center between July 2025 and January 2026. Genetic characterization showed that two specimens harbored an NRAS mutation, and one was NRAS-negative and harbored a BRCA2 mutation. Primary cell cultures were established independently from each patient-derived specimen, and experiments were independently performed using cells derived from different patients. The obtained specimens were washed with Phosphate-buffered saline (PBS) (PB180327, Procell, Wuhan, China) containing 4% penicillin and streptomycin (P/S) (C100C5, Sigma-Aldrich, St. Louis, MO, USA) in sterile culture dishes. The subcutaneous fat was removed carefully with dissecting scissors. Then, tissue samples were cut into 2 mm strips and incubated overnight at 4 °C in 0.25% Dispase II (CD4691, coolaber, Beijing, China) in PBS. After the initial incubation, the epidermis was manually removed from the dermis. The isolated dermis was minced and incubated in 0.25% PBS-diluted collagenase type IV (CC3801G, coolaber) at 37 °C with shaking at 80 rpm for 4 h in a shaking incubator. The suspensions were diluted in DMEM (High glucose) (PM150210, Sigma-Aldrich) and filtered through 70 μm cell strainers. The cells obtained from the dermal components of each lesion were subsequently cultured in complete MelM medium (2201, ScienCell, Carlsbad, CA, USA). To support the melanocytic identity of the isolated primary cells, immunofluorescence staining for the melanocytic markers S100 and MelanA was performed, and representative results are provided in Supplementary Figure S1.

### 2.2. Bulk RNA-Seq and Gene Expression Analysis

GCMN cells (5 × 106 cells/well) were plated in 6-well plates, and after 24 h, they were treated with HL-085 (500 nM, PHLTN191001A, KeChowpharma, Shanghai, China) for 72 h. The concentration of 500 nM tunlametinib was selected based on preliminary CCK-8 dose–response experiments, which identified an IC50 of approximately 500 nM in GCMN cells. Total RNA was extracted using the MJzol Animal RNA Isolation Kit (Majorivd, Shanghai, China) according to the manufacturer’s instructions, followed by purification with the RNAClean XP Kit (Beckman Coulter, Indianapolis, IN, USA) and treatment with RNase-Free DNase Set (QIAGEN, Hilden, Germany) to eliminate genomic DNA contamination. RNA integrity was assessed using an Agilent 2100 Bio-analyzer or Agilent 4200 TapeStation (Agilent Technologies, Santa Clara, CA, US), while RNA concentration and purity were determined using a Qubit 2.0 Fluorometer and NanoDrop ND-2000 spectrophotometer (Thermo Fisher Scientific, Wilmington, DE, USA). Purified RNA was subjected to mRNA enrichment, fragmentation, cDNA synthesis, end repair, adaptor ligation, and PCR amplification to construct sequencing libraries, which were quantified by Qubit 2.0 and quality-checked using the Agilent 4200 TapeStation. Libraries were sequenced on an Illumina NovaSeq 6000 platform (Illumina, Inc., San Diego, CA, USA) with a paired-end 150 bp (PE150) strategy. Clean reads were aligned to the human reference genome (GRCh38) using HISAT2 (version 2.0.4) with default parameters. Gene-level fragment counts were generated using StringTie (version 1.3.0), normalized by the trimmed mean of M values (TMM) method, and converted to FPKM values. Differential expression analysis was performed using edgeR, with multiple testing correction applied to obtain *q*-values. Genes with a *q*-value ≤ 0.05 and |log2 fold change| ≥ 1 were considered differentially expressed. Functional enrichment analyses were conducted using Gene Ontology (GO), Kyoto Encyclopedia of Genes and Genomes (KEGG), and Gene Set Enrichment Analysis (GSEA). RNA sequencing was performed using primary GCMN cells derived from one patient, with three independently treated samples analyzed per condition.

### 2.3. Fontana–Masson Silver Staining

GCMN cells (5 × 106 cells/well) were plated in 6-well plates, and after 24 h, they were treated as indicated in the figures. After 72 h, cells were washed twice with Hank’s Balanced Salt Solution (HBSS) (PB180321, Procell) and fixed with pre-cooled 4% paraformaldehyde (PFA) (BL539A, biosharp, Beijing, China) for 30 min at room temperature. Following fixation, cells were washed again and incubated with Fontana–Masson ammoniacal silver staining solution (G2032, Solarbio, Beijing, China) at 56–60 °C in the dark for 30–60 min, until distinct black granules became visible within the cells. Cells were then rinsed with PBS and treated with sodium thiosulfate (G2032, Solarbio) to remove background silver ions. Stained samples were observed and imaged using a bright-field light microscope. Cells were imaged under identical microscope settings, including magnification, exposure time, light intensity and white balance. Fields were analyzed using a 600 × 600 μm^2^ region of interest (ROI). All images were converted to 8-bit grayscale images in ImageJ (version 1.54p), and dark Fontana–Masson-positive deposits were segmented using a fixed threshold applied uniformly to all images. The Fontana–Masson-positive area and total ROI area were measured, and the positive area percentage was calculated as follows: Area% = Fontana–Masson-positive area/total ROI area × 100%. The same ROI size and threshold range were used for all images to minimize analytical bias.

### 2.4. GCMN Explants

GCMN explant cultures were established with reference to a previously published protocol [17], with minor modifications. Fresh surgical specimens of GCMN were collected, and the subcutaneous tissue was carefully removed. The remaining tissue was cut into approximately 0.5 × 0.5 × 0.5 cm^3^ pieces and maintained in a 1:1 mixture of DMEM and MelM medium for an initial 5-day culture period. The explants were then allocated to untreated control, 100 μM HL-085, and 200 μM HL-085 treatment groups and cultured for an additional 5 days. At the end of treatment, the explants were harvested, rinsed with PBS, fixed in 4% paraformaldehyde, and processed for paraffin embedding. The ex vivo explant experiments were performed using GCMN tissue obtained from one NRAS-mutant patient. A total of nine tissue explants were prepared from this single donor and randomly allocated into three treatment groups, with three explants per group. The 5-day treatment duration was selected with reference to the previously published explant protocol [17]. For dose selection, 50 μM HL-085 was initially chosen based on the concentration range reported in the referenced study, and preliminary experiments were subsequently performed using 10, 25, 50, 100, and 200 μM HL-085. Based on the observed responses in these preliminary experiments, 100 and 200 μM were selected for the final explant experiments presented in this study.

### 2.5. Immunohistochemistry

For immunohistochemical staining, formalin-fixed, paraffin-embedded tissue sections were first deparaffinized with xylene (X820585, Macklin, Shanghai, China) and rehydrated through graded ethanol solutions (E809056, Macklin). Antigen retrieval was carried out in citrate buffer (pH 6.0) at 98 °C for 30 min. After cooling and PBS washing, endogenous peroxidase activity was quenched with 3% H_2_O_2_ for 15 min. To reduce nonspecific binding, the sections were incubated with 5% normal goat serum (SL038, Solarbio) in PBS for 1 h. The sections were incubated overnight at 4 °C with a primary antibody against MelanA (ab51061, Abcam, Cambridge, UK). After washing, the sections were incubated with an HRP-conjugated secondary antibody for 30 min, followed by color development with 3-amino-9-ethylcarbazole (AEC). Finally, nuclei were counterstained with hematoxylin (G1005, Servicebio, Wuhan, China) for 3 min. Apoptotic cells in the explant sections were assessed using a TUNEL assay according to the manufacturer’s instructions. MelanA immunohistochemical staining and TUNEL staining were evaluated qualitatively by visual inspection of the staining density and distribution. Representative images were selected for presentation. No quantitative scoring or statistical analysis was performed for these staining results.

### 2.6. Hematoxylin and Eosin (H&E) Staining

Tissue samples were fixed in 4% paraformaldehyde, embedded in paraffin, and sectioned at a thickness of 4–5 μm. Sections were deparaffinized in xylene and rehy-drated through a graded ethanol series to distilled water. Slides were then stained with hematoxylin to visualize cell nuclei, followed by differentiation and bluing. After rinsing, sections were counterstained with eosin (G1005, Servicebio) to label cytoplasmic and extracellular components. Finally, sections were dehydrated through graded ethanol, cleared in xylene, and mounted with rhamsan gum (WG10004160, Servicebio). Stained sections were examined and imaged using a light microscope.

### 2.7. Western Blot (WB) Analysis

Total protein was extracted using RIPA lysis buffer (pc101, Yamay, Shanghai, China) containing protease inhibitor and phosphatase inhibitor (grf101, Yamay) on ice for 15–20 min. The extracted proteins were separated by sodium dodecyl sulfate–polyacrylamide gel electrophoresis (SDS-PAGE) (GF1820, Genefist; PS105S, Yamay) and subsequently transferred onto polyvinylidene fluoride (PVDF) membranes (FFP22, Beyotime, Shanghai, China). The membranes were blocked with 5% bovine serum albumin (BSA) solution (4240GR100, BioFroxx, Einhausen, Germany) for 2 h at room temperature and then incubated with the corresponding primary antibodies overnight at 4 °C. Following washing with Tris-buffered saline with Tween 20 (TBST) (PS103S, Yamay), the membranes were incubated with the appropriate secondary antibodies at room temperature for 2 h according to the host species of the primary antibodies. Protein signals were developed using the Omni-ECL™ Femto Light Chemiluminescence Kit (SQ201, Yamay) and visualized with a ChemiDoc MP Imaging System (Tanon-4600, Shanghai, China). Band intensities were quantified using ImageJ software. Target protein expression levels were normalized to β-actin or GAPDH and expressed relative to the untreated control, which was assigned a value of 1. Information on the primary antibodies used in this study is provided in Supplementary Table S1.

### 2.8. Cell Viability Assay

GCMN cells (1 × 10^5^ cells/well) were plated in 96-well plates, and after 24 h, they were treated with HL-085 and assayed for viability after 72 h with a CCK8 kit (C0038, beyotime) according to the manufacturer’s instructions.

### 2.9. Apoptosis Assays

Cells were seeded in 6-well plates at 5 × 10^5^ cells/well and treated with HL-085 for 72 h. Apoptosis was assessed using an Annexin V-FITC/PI apoptosis detection kit (E-CK-A217, Elabscience, Wuhan, China) according to the manufacturer’s instructions. Briefly, culture supernatants containing floating cells were first collected, and adherent cells were gently washed with PBS and detached using EDTA-free trypsin (PB180228, Procell) at 37 °C for 3–5 min. The detached cells were combined with the previously collected supernatants and centrifuged at 1500 rpm for 5 min. After washing once with PBS, the collected cells were resuspended in 100 μL of 1× binding buffer. The cells were then incubated with 5 μL of Annexin V-FITC and 5 μL of PI reagent (50 μg/mL) for 15 min at room temperature in the dark. Later, the stained cells were analyzed by flow cytometry. Data were analyzed using flowjo software, version 10.8.1.

### 2.10. Cell-Cycle Synchronization and Analysis

Cell cycle distribution was analyzed using the Cell Cycle Detection Kit (AC101-01/02, Vazyme, Nanjing, China) according to the manufacturer’s instructions. Briefly, cells were harvested after the indicated treatments, washed twice with cold PBS, and fixed in 70% ice-cold ethanol at 4 °C for at least 2 h (or overnight). After fixation, cells were washed to remove residual ethanol. Subsequently, cells were stained with staining solution at room temperature in the dark for 30 min. DNA content was analyzed using a flow cytometer, and cell cycle distribution (G0/G1, S, and G2/M phases) was quantified using modfit software, version 5.0.

### 2.11. Statistical Analysis

The data are presented as the mean ± standard deviation (SD). Primary GCMN cells were independently established from three patients. CCK-8 assays, Western blotting, and flow cytometry were performed using cells from all three patients as three independent biological replicates, with three technical replicates for each biological replicate. RNA sequencing was performed using cells derived from one patient, with three technical replicates per group. Explant experiments were conducted using tissue from a single NRAS-mutant patient, with three explants per treatment group. These results were evaluated qualitatively without statistical analysis. Student’s *t*-test was used to assess differences between two groups. Statistical analyses and graphical plotting for the study were performed using GraphPad Prism 10.1.2. *p* < 0.05 was considered statistically significant and denoted as follows: * *p* < 0.05, ** *p* < 0.01, *** *p* < 0.001, **** *p* < 0.0001.

## 3. Results

### 3.1. HL-085 Inhibited Melanogenesis in GCMN Cells

**Figure 1:**
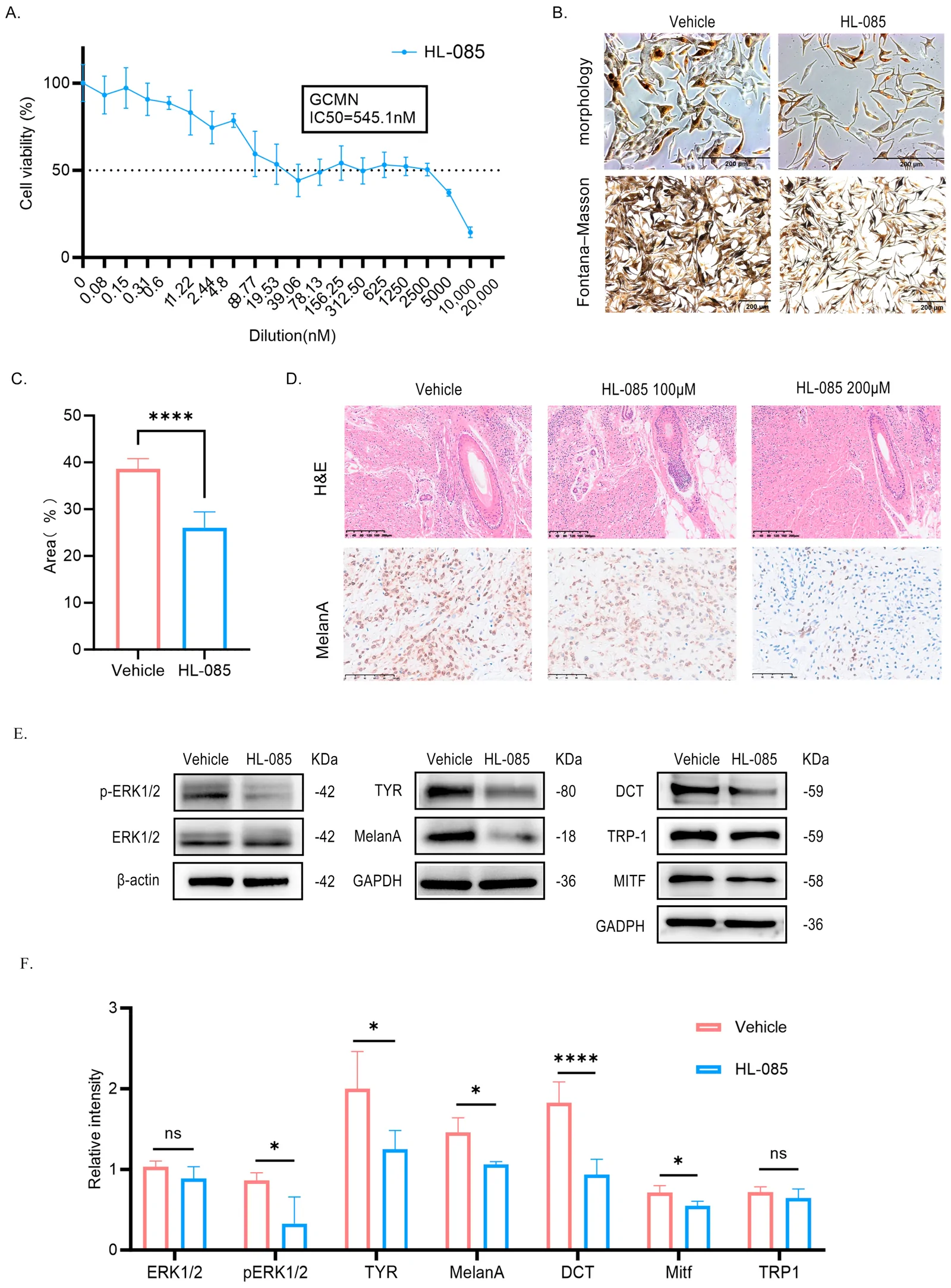
(**A**). CCK8 assay evaluating the efficacy of HL-085 in GCMN cells. (**B**). Cultured GNCs were treated with HL-085 (500 nM) for 72 h. Cell morphology was examined, and melanin deposition was evaluated by Fontana–Masson staining. (**C**). Quantification of Fontana–Masson-positive area. (**D**). HE staining and MelanA immunohistochemical staining (cytoplasmic) of GCMN explants after receiving HL-085 treatments (100 μM, 200 μM, 5 days). (**E**). Cultured GNCs were treated with HL-085 (500 nM) for 72 h, and WB analysis was done for ERK1/2, pERK1/2, TYR, MelanA, DCT, MITF and TRP1. (**F**). Quantitative analysis of ERK/MAPK signaling and melanogenesis-related protein expression after HL-085 treatment (ns, *p* ≥ 0.05, * *p* < 0.05, **** *p* < 0.0001).

The CCK-8 assay showed that HL-085 reduced the metabolic activity of GCMN cells in a dose-dependent manner, with an IC50 of approximately 545.1 nM. (Figure 1A). The GCMN cells were treated with HL-085 (500 nM) for 72 h. Under light microscopy, untreated GCMN cells exhibited a spindle-shaped morphology with abundant cytoplasm, whereas drug-treated cells appeared more elongated, less spread, and displayed thinner cytoplasm with lighter staining and reduced or dispersed pigment-like granules. Subsequently, Fontana–Masson staining showed reduced melanin-containing deposits in HL-085-treated GCMN cells, as shown in the figure (Figure 1B). ImageJ-based quantification revealed that the percentage of Fontana–Masson-positive area was significantly lower in drug-treated cells than in control cells (Figure 1C). In our GCMN explants, HL-085 significantly reduced nevus cell density and pigmentation. MelanA staining was also decreased following drug treatment (Figure 1D). As expected, Western blot analysis showed that the phosphorylation of ERK1/2 was significantly decreased in GCMN cells (Figure 1E,F). MITF serves as a central transcriptional regulator of melanocytic lineage and differentiation, transcriptionally controlling multiple melanogenesis- and melanosome-related genes, including TYR, TRP1, DCT, and MelanA). Among these, TYR is the rate-limiting enzyme for melanin synthesis, TRP1 and DCT participate in the melanogenic process, and MelanA reflects melanocytic differentiation and melanosome biogenesis [23,24,25,26], we further examined the expression of these proteins to assess alterations in the melanocyte differentiation–melanogenesis axis following HL-085 treatment. The results showed that, with the exception of TRP1, all other melanogenesis-related proteins were significantly downregulated in GCMN cells after HL-085 treatment (Figure 1E,F).

### 3.2. HL-085 Induced a Distinct Gene Expression Pattern in GCMN Cells

**Figure 2:**
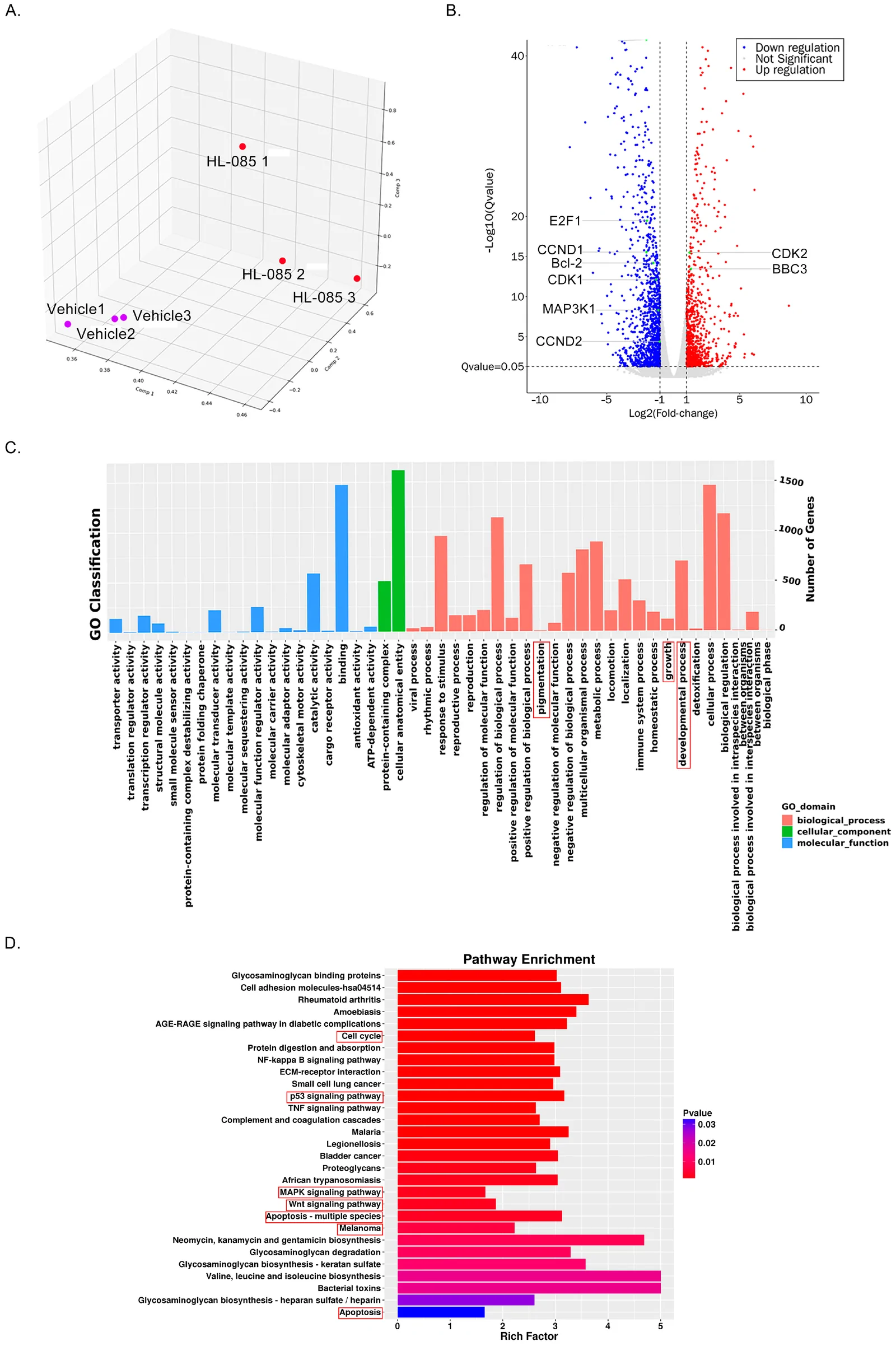
(**A**). PCA of the transcriptome of RNA-seq data from GCMN cells with DMSO or HL-085 treatment. (**B**). Volcano plot of the transcriptome of RNA-seq data from GCMN cells with DMSO or HL-085 treatment. (**C**). The top 50 enriched GO terms among DEGs by functional classification. (**D**). The top 160 pathway terms enriching DEGs in Kyoto Encyclopedia of Genes and Genomes (KEGG) analysis (partial).

The transcriptomic effects of HL-085 were further investigated in human primary GCMN cells. Cells were treated with 500 nM HL-085 for 72 h at 37 °C, followed by RNA sequencing analysis. Principal component analysis (PCA) was first performed to evaluate the overall transcriptional differences between the DMSO and HL-085 groups, as well as the reproducibility among biological replicates. The two groups formed clearly separated clusters, while samples within the same group clustered closely, indicating distinct treatment-induced transcriptional profiles and satisfactory intra-group reproducibility (Figure 2A). Differential gene expression analysis was then performed to identify genes affected by HL-085 treatment. As shown in the volcano plot, HL-085 treatment induced substantial changes in gene expression compared with the DMSO group (Figure 2B). Notably, several genes associated with cell-cycle progression and survival, including *CCND1*, *CCND2*, *E2F1*, *Bcl-2*, *CDK1*, and *MAP3K1*, were downregulated, whereas apoptosis- and cell-cycle-related genes such as *CDK2* and *BBC3* were upregulated. In total, 1324 genes were downregulated and 924 genes were upregulated following HL-085 treatment, using the criteria of |log2 fold change| ≥ 1 and *q*-value ≤ 0.05. To further characterize the biological implications of these transcriptional changes, GO enrichment analysis was performed. The DEGs were significantly enriched in biological processes related to reproduction, pigmentation, growth, and development, suggesting that HL-085 treatment broadly affected pigment regulation, growth control, and developmental programs in GCMN cells (Figure 2C). KEGG pathway analysis further categorized the DEGs into 276 signaling pathways. Among the highly enriched pathways, the apoptosis, cell cycle, and MAPK signaling pathways were prominently represented (Figure 2D). Based on these transcriptomic findings, subsequent analyses focused primarily on the regulatory effects of HL-085 on cell-cycle progression and apoptosis.

### 3.3. HL-085 Induced Apoptosis in GCMN Cells

**Figure 3:**
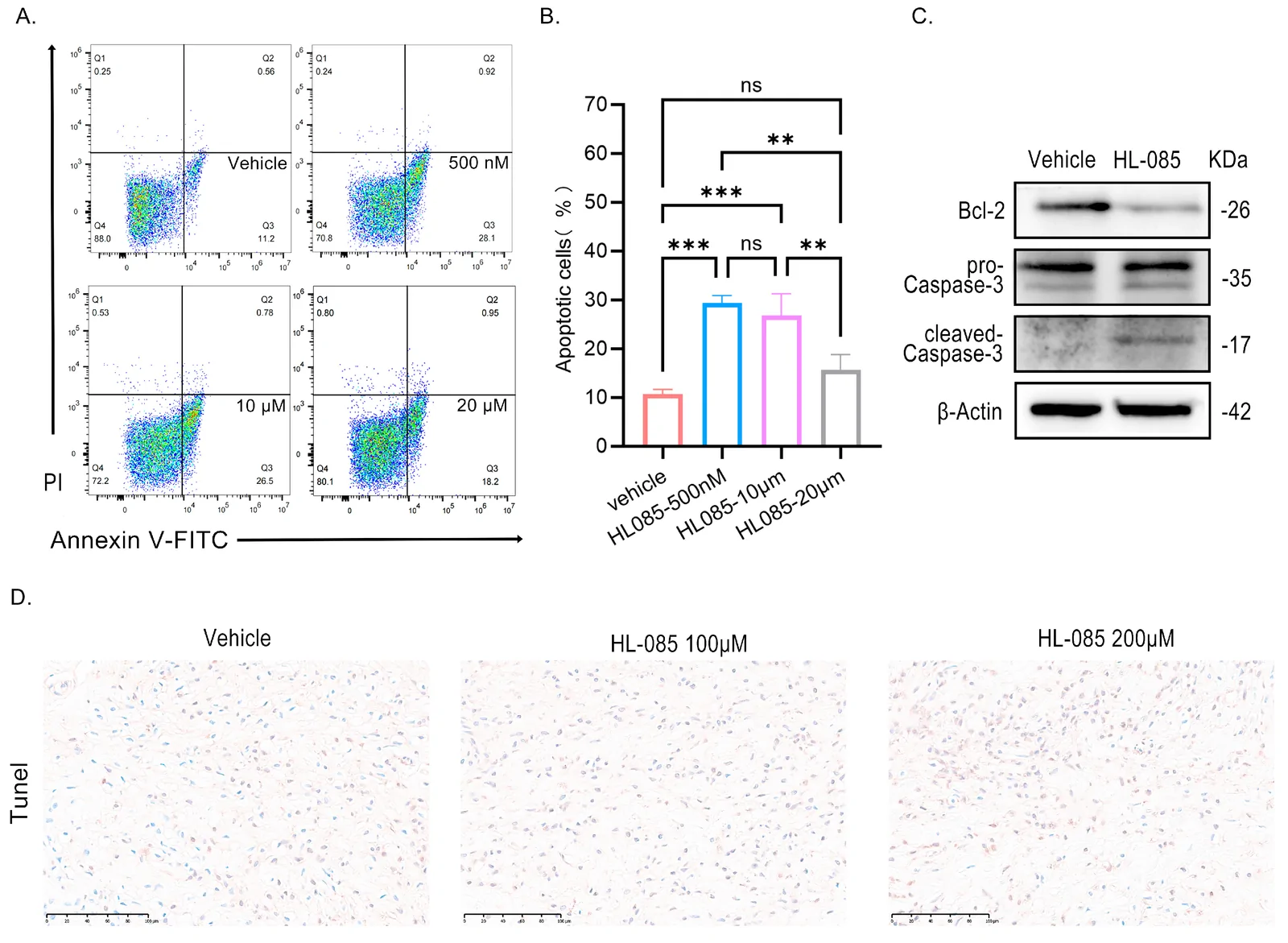
(**A**). Flow cytometry analysis of apoptotic activity in GCMN cells after 72 h of treatment with DMSO, HL-085 (500 nM), HL-085 (10 μM) and HL-085 (20 μM). (**B**). Quantitative analysis of apoptotic cells (ns, *p* ≥ 0.05, ** *p* < 0.01, *** *p* < 0.001). (**C**). Cultured GNCs were treated with HL-085 (500 nM) for 72 h, and WB analysis was done for Bcl-2, pro-Caspase-3 and cleaved-Caspase-3. (**D**). TUNEL staining of GCMN after HL-085 treatment (100 μM, 200 μM, 5 days).

Flow cytometric analysis showed that HL-085 treatment predominantly induced early apoptosis in GCMN cells. Quantification of total apoptotic cells demonstrated a significant increase in apoptosis after treatment with 500 nM and 10 μM HL-085 compared with the untreated control, with no significant difference between these two concentrations. In contrast, the 20 μM group did not differ significantly from the control, and showed significantly lower apoptosis than both the 500 nM and 10 μM groups (Figure 3A,B). To assess apoptosis-related changes, immunoblotting revealed an increase in cleaved caspase-3 and a concomitant decrease in Bcl-2 expression following HL-085 treatment, suggesting an association between HL-085 treatment and apoptotic responses in GCMN cells (Figure 3C). Additionally, a Terminal dUTP Nick End Labeling (TUNEL) assay was performed on GCMN explants. TUNEL staining revealed an increase in TUNEL-positive cells following HL-085 treatment (Figure 3D).

### 3.4. HL-085 Induced G1-S Cell Cycle Arrest of GCMN Cells

**Figure 4:**
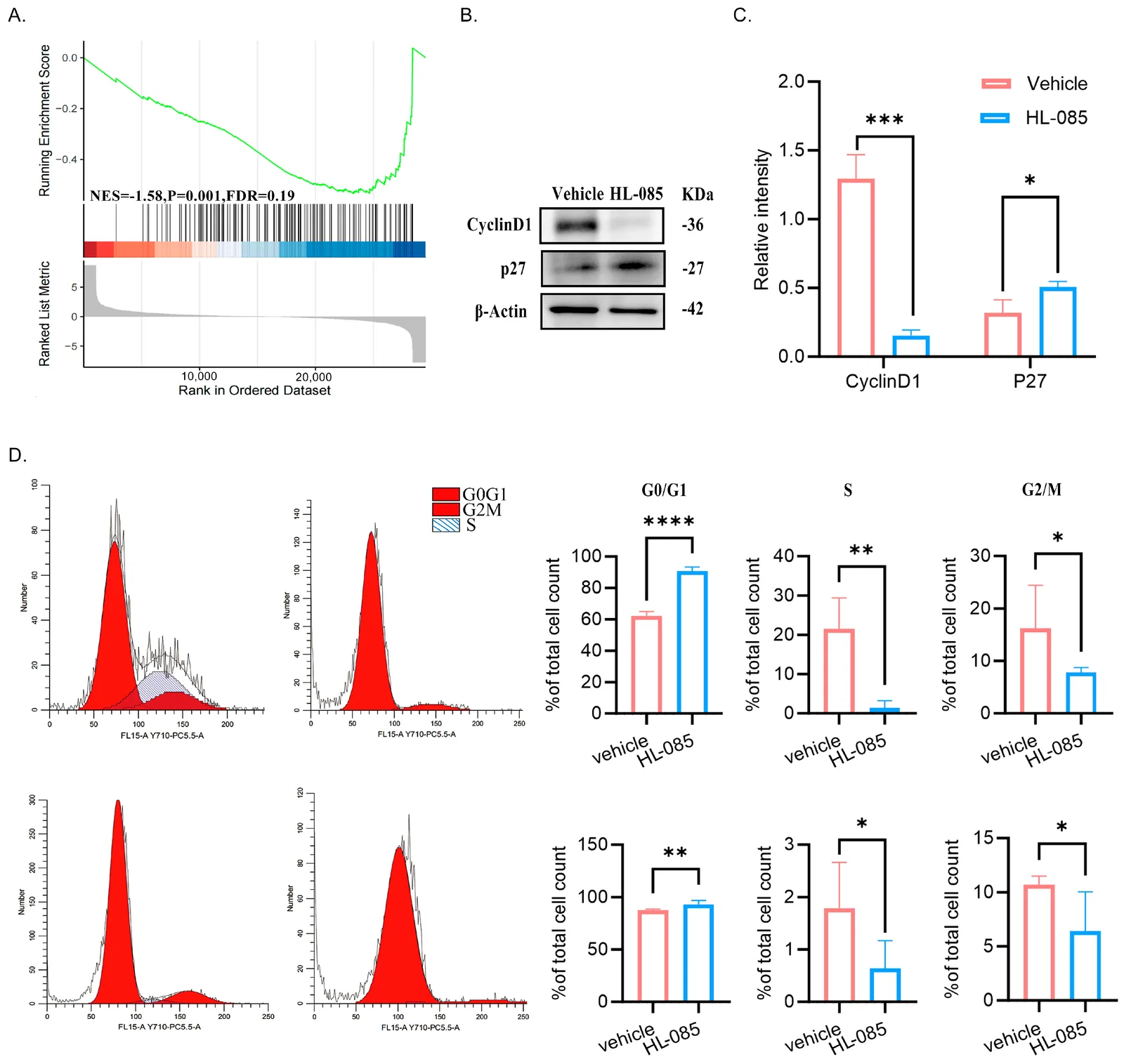
(**A**). GSEA of G1 phase and G1/S transition of HL-085 and DMSO in GCMN cell (|NES| > 1; FDR < 0.25; *p* < 0.05). (**B**). Cultured GNCs were treated with HL-085 (500 nM) for 24 h, and WB analysis was done for CyclinD1 and p27. (**C**). Quantitative analysis of CyclinD1 and p27 signaling and melanogenesis-related protein expression after HL-085 treatment. (**D**). Cell cycle analysis of GCMN cells after DMSO and HL-085 treatment for 24 h and 48 h by flow cytometry using PI staining (* *p* < 0.05, ** *p* < 0.01, *** *p* < 0.001, **** *p* < 0.0001).

According to our RNA-seq data, Gene Set Enrichment Analysis (GSEA) demonstrated a trend toward negative enrichment of gene sets associated with the G1 phase and G1-S transition in the HL-085 treatment group compared with the DMSO group (NES = −1.58, FDR = 0.19), suggesting attenuated G1/S cell cycle progression at the transcriptional level (Figure 4A). We then detected the expression levels of cell cycle-related proteins. The data showed that Cyclin D1 was significantly attenuated, whereas the cell cycle-inhibitory protein p27 was markedly increased in GCMN cells after 24 h of HL-085 treatment compared with control cells (Figure 4B,C). Functionally, we performed PI staining assays at three time points (24 h and 48 h) in GCMN cells. Cells were synchronized to the G1 phase using serum starvation prior to the experiments. Cell cycle analysis revealed that G1-S arrest was already observed after 24 h of HL-085 treatment. However, this effect gradually diminished at 48 h and 72 h, and no longer reached statistical significance (Figure 4D).

## 4. Discussion

GCMN represents not only a cosmetic burden but also a clinically significant disorder associated with neurocutaneous melanosis and an increased lifetime risk of malignant transformation into melanoma [27]. Surgical excision remains the mainstay of treatment yet is limited by incomplete lesion removal, significant scarring, and the impracticality of complete resection in extensive cases [28]. In recent years, MEK inhibitors have been explored as targeted therapies for GCMN harboring activating NRAS mutations. Despite early clinical observation of partial symptomatic improvement, including reduced pruritus or lesion softening, regression of pigmentation and lesion size has been limited [9,10,11,29]. Moreover, even when reductions in nevus burden, pigmentation, or lesion size are achieved, these changes should not be interpreted as evidence of reduced melanoma risk, as the preventive effect of MEK inhibition against malignant transformation remains unestablished. Concerns over drug toxicity and acquired resistance continue to pose substantial barriers to long-term use, underscoring the considerable clinical importance of developing more potent and well-tolerated inhibitors [30].

In this study, we evaluated HL-085, a novel and highly selective MEK inhibitor that has shown clinical efficacy in NRAS-mutant melanoma and has been included in the CSCO melanoma treatment guidelines. We demonstrated that HL-085 significantly reduced pigmentation-related signals and inhibited proliferation in primary GCMN cells. Mechanistically, these effects correlated with G1–S cell cycle arrest and increased early apoptosis. Taken together, these findings provide preclinical proof-of-concept for the potential application of HL-085 in GCMN, rather than evidence of clinical efficacy, and support further translational and clinical investigation. Our findings are consistent with prior studies evaluating MEK inhibition in GCMN models [9,10,17]. Trametinib was first reported to suppress proliferation and induce apoptosis in CMN-derived cells. Similarly, MEK inhibition reduced melanocytic proliferation in NRAS-mutant CMN models. More recently, MEK inhibitor treatment was shown to decrease expression of melanocytic lineage markers, including MelanA and SOX10, in vitro. However, these studies did not comprehensively assess melanogenic function or delineate cell cycle dynamics. In contrast, our observation demonstrated a marked reduction in melanin production along with clear G1–S arrest, offering further mechanistic insight into how MEK inhibition modulates CMN cellular behavior beyond merely suppressing proliferation.

Of note, tunlametinib treatment markedly downregulated MITF and its downstream melanogenesis-associated proteins, including TYR, DCT, and MelanA in GCMN cells, suggesting that tunlametinib may attenuate melanocytic differentiation and melanogenic capacity via inhibition of the MAPK–ERK signaling pathway. However, these reductions should be interpreted with caution, as they may also partly reflect decreased cellular activity, treatment-related cell loss, or changes in cellular composition following tunlametinib exposure. TRP1 expression was not synchronously reduced, suggesting that melanogenesis-related molecules exhibit differential sensitivity to MEK inhibition. Although TRP1 is generally considered as an MITF-regulated melanogenic gene, previous studies have shown that its expression can remain relatively stable in certain melanoma contexts and may even be regulated independently. Notably, TRP1 expression was reported to remain unchanged in *BRAF^V600^* mutated melanoma cells resistant to vemurafenib or trametinib [31]. Thus, the absence of significant TRP1 downregulation in our study may point to a regulatory mechanism distinct from that governing TYR and DCT in GCMN cells. Mechanistically, TYR and DCT may more directly reflect the overall suppression of the melanogenic enzyme system following MITF downregulation, whereas TRP1 may also be influenced by other transcription factors, epigenetic states, protein stability, or melanosome maturation status. Previous studies have established MITF as a master regulator of melanocyte differentiation and melanogenesis, exerting transcriptional control over multiple pigmentation-related genes, including TYR, TRP1, and DCT [32]. Nevertheless, the magnitude and timing of responses among individual MITF target genes may vary depending on cellular context. Thus, preserved TRP1 expression does not necessarily contradict the inhibitory effect of tunlametinib on melanogenesis. Rather, it may suggest that GCMN cells retain partial melanosomal structure or melanocytic lineage features after MEK inhibition. However, these protein-level alterations should be considered as supportive evidence of altered melanocytic function rather than definitive proof of reduced melanin synthesis, and further functional assays are required to confirm the direct effects of tunlametinib on melanogenesis.

Despite not revealing significant enrichment of melanogenesis-related pathways in our earlier RNA-seq analysis, clear reductions in melanin production and melanogenic protein levels were observed at both the phenotypic and protein levels. This discrepancy may be attributed to post-transcriptional regulation. It is well established that melanogenesis is heavily controlled at translational and post-translational levels, involving MITF phosphorylation, ubiquitination, and proteasomal degradation [23,32]. Moreover, MAPK signaling may regulate melanogenesis at both the transcriptional and post-transcriptional/post-translational levels of melanogenic enzymes. For instance, p38 MAPK has been shown to modulate pigmentation via tyrosinase ubiquitination and proteasomal degradation [33], suggesting that functional suppression may occur without robust transcriptional changes. Similar studies have shown that, in the context of MAPK inhibitor treatment or acquired resistance, transcriptomic alterations may not fully correspond to protein-level or phenotypic changes [34]. Future studies should experimentally investigate these potential post-transcriptional and post-translational regulatory mechanisms, including analyses of mRNA stability, translational regulation, protein phosphorylation, ubiquitination, and proteasomal degradation of key melanogenic regulators.

HL-085 induced a pronounced G0/G1 accumulation with reductions in S and G2/M phases at 24 h, consistent with G1/S cell-cycle arrest. However, this effect was transient and became markedly attenuated at 48 and 72 h. Consistently, several studies have shown that MEK inhibitors induce G1-S cell-cycle arrest in melanoma and other BRAF-mutant malignancies, including non-small cell lung cancer [35,36]. Notably, this effect was most evident at 24 h and became less pronounced with prolonged exposure. This finding may be explained by the dynamic nature of the cell population. Cell-cycle analysis captures only surviving cells. Consequently, with prolonged MEK inhibition, sensitive cells may undergo apoptosis or other forms of death, leaving behind a population of relatively tolerant or quiescent cells, which reduces the observed difference between G1 and S phases. In addition, sustained MEK inhibition does not necessarily maintain permanent G1 arrest. Channathodiyil et al. [35] showed that some selumetinib-treated cells can escape G1 arrest and re-enter the cell cycle during continuous exposure. Feedback activation of compensatory pathways may also contribute. MEK inhibition can activate survival signals such as PI3K–AKT through RTK-mediated feedback. Therefore, tunlametinib appears to induce an early but transient G1/S arrest, while prolonged exposure may permit adaptive survival, cell-cycle re-entry, or compensatory signaling, thereby reducing the apparent arrest phenotype over time. Future studies should experimentally investigate these possibilities through time-resolved analyses of cell-cycle progression and apoptosis, lineage-tracing or cell-recovery experiments, and assessment of compensatory signaling pathways, such as PI3K–AKT activation, to determine whether prolonged tunlametinib exposure leads to cell-cycle re-entry, selective survival of tolerant cells, or adaptive responses.

Notably, the apoptotic response to HL-085 was not dose-dependent, as the apoptotic fraction was lower in the 20 μM group than in the 500 nM and 10 μM groups. This unexpected finding may reflect a limitation of single-time-point Annexin V/PI analysis rather than a true reduction in cytotoxicity. Annexin V/PI staining captures a dynamic process in which cells progress from early apoptosis to late apoptosis or secondary necrosis, and excessive drug exposure may accelerate this transition beyond the early apoptotic window. Moreover, severe cytotoxicity in adherent cells may cause detachment and loss of fragile dying cells during sample preparation, leading to underestimation of the apoptotic population. Therefore, the 20 μM result should be interpreted cautiously, and time-course analysis or complementary cell death assays would be required to further clarify this phenomenon.

Taken together, our study demonstrates that HL-085 suppresses proliferation and melanogenesis in GCMN cells, partly through induction of G1–S cell cycle arrest and promotion of early apoptosis. These findings provide preclinical evidence supporting HL-085 as a potential targeted therapeutic option for GCMN. Nevertheless, several limitations need to be acknowledged. A limitation of our work is that it primarily relied on primary GCMN cells and ex vivo explant cultures, while in vivo validation remains lacking. Furthermore, long-term resistance mechanisms and safety profiles remain yet to be evaluated.

Several limitations should be acknowledged. First, the number and genetic diversity of the patient-derived GCMN models were limited. Although primary cells from three independent patients were included, RNA sequencing and the explant model were derived from a single NRAS-mutant donor. Therefore, genotype-dependent responses to tunlametinib could not be systematically evaluated. Future studies should include a larger panel of genetically characterized GCMN models with defined NRAS, BRAF, and other MAPK-pathway alterations. Meanwhile, although Melan-A and S100 staining supported the melanocytic identity of the isolated cells, more comprehensive phenotypic and genotypic validation remains warranted. Second, normal melanocytes, normal skin controls, and melanoma cell comparators were not comprehensively included, limiting assessment of whether tunlametinib preferentially or specifically affects GCMN cells. Future studies incorporating appropriate normal and malignant melanocytic controls will be important to evaluate treatment selectivity. Third, the pigmentation-related findings should be interpreted cautiously. Fontana–Masson staining reflects melanin-containing deposits but does not directly quantify melanogenesis, and the observed reductions in pigmentation, Melan-A staining, and pigmentation-associated proteins may partly reflect reduced cell number or altered cellular composition. Direct measurements of melanin content and melanogenic enzyme activity will therefore be required to establish functional effects on melanin synthesis. In addition, MelanA immunohistochemistry and TUNEL staining were evaluated qualitatively without formal quantitative scoring or statistical analysis, and future studies should incorporate standardized quantitative image analysis. Finally, the apoptosis-related evidence remains limited, as the increase in cleaved caspase-3 was modest, cleaved PARP was not assessed, and reduced Bcl-2 expression alone cannot establish a specific pro-apoptotic mechanism. Additional apoptosis markers and complementary functional assays are needed to further validate these findings. A minor limitation also concerns the graphical presentation of the Annexin V/PI data, as some low-fluorescence events appear close to the display boundary, although these events were retained in the original FCS data and included in the analysis. More importantly, the present findings represent preclinical proof-of-concept and should not be interpreted as evidence of clinical efficacy or melanoma-preventive benefit.

Future studies should further define the mechanisms underlying the observed biological responses to tunlametinib. Key RNA-seq findings should be independently validated at both the mRNA and protein levels, with particular attention to biologically relevant targets. The potential post-transcriptional and post-translational mechanisms underlying transcript–protein discrepancies should also be examined, including mRNA stability, translational regulation, phosphorylation, ubiquitination, and proteasomal degradation. In addition, time-resolved cell-cycle and apoptosis analyses, cell-tracing or recovery experiments, and assessment of compensatory pathways such as PI3K–AKT will be important to determine whether the transient G1/S arrest reflects subsequent cell-cycle recovery, selective survival of tolerant cells, apoptosis, or adaptive feedback. Dose–response studies at non-cytotoxic concentrations should also be performed to better establish the temporal and causal relationships among MEK/ERK inhibition, cell-cycle regulation, apoptosis, and pigmentation-related changes. Finally, future studies could establish an in vivo patient-derived GCMN xenograft model by inoculating GCMN cells into immunocompromised mice. Such a model would enable further evaluation of the in vivo efficacy, durability of response, safety, and translational potential of tunlametinib in GCMN.

## 5. Conclusions

Functionally, tunlametinib was associated with reduced pigmentation-related signals, suppressed cellular proliferation, increased apoptosis, and transient G1/S cell-cycle arrest in GCMN cells. These findings suggest that MEK inhibition affects multiple biological processes relevant to GCMN, although the causal relationships among cell-cycle changes, apoptosis, and pigmentation-related effects remain to be established. Given the limited number of patient-derived models and the preclinical nature of the present study, further investigations are required to validate these findings in additional genetically characterized GCMN models and to evaluate the safety, efficacy, and durability of tunlametinib in vivo.

## Data Availability

The original contributions presented in this study are included in the article. Further inquiries can be directed to the corresponding author.

## Supplementary Materials

The following supporting information can be downloaded at: https://www.mdpi.com/article/10.3390/cancers18193166/s1, Figure S1: Immunofluorescence staining of MelanA and S100 in primary GCMN cells; Table S1: Antibodies used for immunofluorescence..

## Abbreviations

The following abbreviations are used in this manuscript:

GCMN	Giant congenital melanocytic nevus
GO	Gene Ontolog
KEGG	Kyoto Encyclopedia of Genes and Genomes
GSEA	Gene Set Enrichment Analysis
mAb	monoclonal antibody
TUNEL	Terminal dUTP Nick End Labeling
TMM	trimmed mean of M values
PFA	paraformaldehyde (PFA)
