# Supplementary material for: Tunlametinib Suppresses Melanogenesis Induces Cell Cycle Arrest and Apoptosis in Giant Congenital Melanocytic Nevus Cells
Source: Cancers (Basel). 2026 Oct 1;18(19):3166. doi: 10.3390/cancers18193166 (PMC13664429; doi:10.3390/cancers18193166)
Supplement: Supplementary file 1 [file cancers-18-03166-s001.zip › cancers-4484220-supplementary.pdf]

Table S1. Antibodies used for immunofluorescence.

Antibody Name	Manufacturer	Catalog No.
β-actin Rabbit monoclonal antibody(mAb)	Immunoway	YM8343
GADPH Rabbit mAb	yammy	LF211-HRP
Bcl-2 Rabbit mAb	Immunoway	YM8319
Caspase-3 Rabbit mAb	Immunoway	YM8058
Cleaved Caspase-3 Rabbit mAb	Immunoway	YM8294
p-ERK1/2 Rabbit mAb	Immunoway	YM8452
ERK1/2 Rabbit mAb	Immunoway	YM8336
DCT Rabbit mAb	Immunoway	YT5849
MITF Rabbit mAb	Immunoway	YM8062
Tyrpsinase Mouse mAb	Immunoway	YM4942
MelanA Rabbit mAb	Abcam	ab51061
TRP1 Rabbit pAb	Immunoway	YN8781
p27 KIP 1 Mouse mAb	Immunoway	YM4947
S100 Rabbit mAb	Abcam	ab52234
Cyclin D1 Rabbit mAb	Immunoway	YM8149
Goat Anti Mouse IgG（H+L)（HRP）	Immunoway	RS0001
Goat Anti Rabit IgG（H+L)（HRP）	yammy	LF102
Alexa Fluor®488 donkey anti-rabbit lgG(H+L)	ThermoFisher	A21207

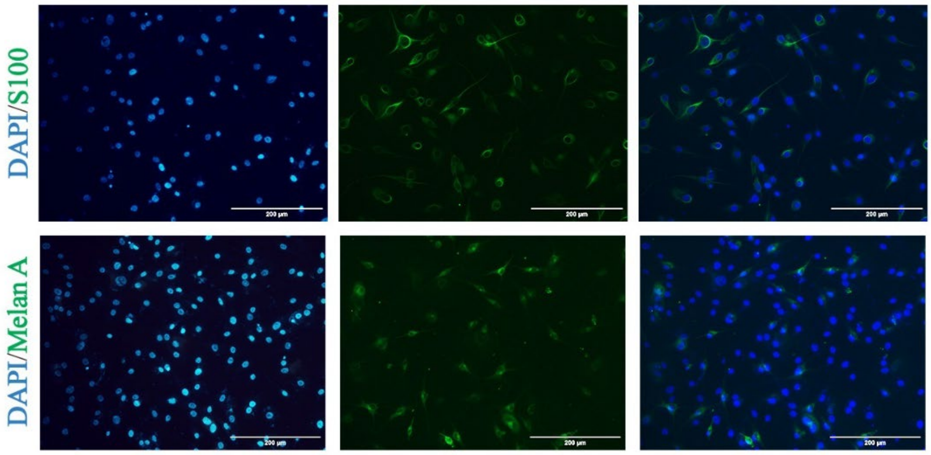

Supplymentry Figure S1. Immunofluorescence staining of MelanA and S100 in primary GCMN cells.
